# Preservation of female fertility in humans and animal species

**DOI:** 10.21451/1984-3143-AR2018-0089

**Published:** 2018-08-17

**Authors:** Helen Mary Picton

**Affiliations:** Reproduction and Early Development Research Group, Discovery and Translational Science Department, Leeds Institute Of Cardiovascular and Metabolic Medicine, School of Medicine, University of Leeds, UK.

**Keywords:** cryopreservation, fertility preservation, oocyte, ovary

## Abstract

A detailed understanding of the cryobiology of gametes and complex tissues has led to the development of methods that facilitate the successful low temperature banking of isolated mature human oocytes, or immature oocytes *in situ* within fragments of human ovarian cortex. Although many outstanding research challenges remain to be addressed, the successful development of new treatments to preserve female fertility for a range of clinical indications has largely been underpinned by the conduct of extensive, fundamental research on oocytes and ovarian tissues from a number of laboratory and commercially important farm species. Indeed, the most recent evidence from large animals suggests that it is also possible to cryopreserve intact whole ovaries along with their supporting vasculature for later auto-transplantation and restoration of natural fertility. This review will explore how the methods developed to preserve human oocytes and ovarian tissues can now be used strategically to support the development of conservation strategies aimed at safeguarding the genetic diversity of commercially important domestic animals and also of preserving the female germplasm for wild animals and endangered species.

## Introduction

In the last few decades, advances in cryobiology have been combined with the development of new assisted reproduction technologies (ARTs) and used as a means to cryopreserve the structural integrity and biological function of key reproductive cells. Translation of these research advances has resulted in the development of the capacity to cryopreserve and long-term store isolated gametes, embryos, complex gonadal tissues and even whole reproductive organs in humans and laboratory species as well as commercially important farm animal breeds and a limited number of exotic or endangered species. Indeed, the issue of fertility preservation is particularly relevant in animals as over the last two decades, some 300 of 6000 farm animal breeds have become extinct and a further 1350 domestic breeds are being threatened with extinction as a result of aggressive animal breeding strategies using limited genetic stocks of animals of high merit for a range of economically valuable traits ( Taberlet *et al*., 2008 ).

The standard method of fertility preservation in males whether humans or animals is the cryopreservation and storage of sperm (for review see Ehmcke and Schlatt, 2008 ). Similarly, since the first successful cryopreservation of embryos in laboratory species in the early 1970s ( Whittingham *et al*., 1972 ) thousands of animal and human offspring have been born following embryo cryopreservation. With regard to oocytes, the recent development of improved methods and devices for the vitrification of isolated cells has increased the success rates of metaphase II (MII), oocyte preservation in humans ( Rienzi *et al*., 2017 ). This technological advance has only had limited application to the oocyte preservation in animals due to a general lack of basic understanding of oocyte biology in different target species ( Comizzoli, 2017 ). While sperm, oocytes and embryo preservation using ART are clearly effective, recent research focus has shifted to the preservation of gametes *in situ* either within fragments of ovarian and testicular tissue or following whole organ cryopreservation. Indeed, the banking of gonadal tissue is particularly valuable as a means of preserving fertility as in the case of the ovary the banked tissue may contain thousands of the earliest staged primordial follicles and oocytes. In contrast testicular tissue cryopreservation facilitates the banking of spermatogonial stem cells that can subsequently can be harvested and used to repopulate the testis and restart spermatogenesis ( Picton *et al*., 2015 ). Although no standardized protocols exist for fertility preservation in any species, the preservation of gonadal tissues offers near unlimited potential for fertility preservation and restoration in humans as well as domestic and wild animal species. This review aims to provide an overview of the recent advances in human oocyte and ovarian tissue banking and how these clinical reproductive technologies can be used to support fertility conservation strategies in animals.

## Cryobiology and fertility preservation

Cryopreservation is a multi-step procedure that requires that cells tolerate exposure to: molar concentrations of penetrating and non-penetrating cryoprotective agents (CPAs); cooling to subzero temperatures; and either removal or conversion of the greater majority of liquid water within the cells into a solid state. The reverse occurs during thawing or warming. Any of these elements can inflict damage on the cells to be preserved although the level and nature of the damage is dependent on the cryobiological properties of each individual cell type. With regards to the cryopreservation of complex tissues containing multiple cell types, such as the ovary, the success of cryopreservation is dependent on the need to balance the freezing optima for a range of different cell types which are influenced by cell number and size and, in the case of oocytes, maturational status, as well as the requirement to preserve the structural integrity of the tissue. Furthermore, the nature of the cryopreservation methodology, whether slow freezing or vitrification, the cooling and warming rates and the containment vessel used for tissue preservation and storage will all influence the efficacy of the preservation method and hence impact on the subsequent viability of the preserved tissue. Finally, the unique biological properties of oocytes challenge our potential to freeze-store these important cells. Mammalian oocytes are very large cells of approximately 120 µm diameter with a small surface area to volume ratio and high lipid and water content. The latter confers a high sensitivity to chilling injury and intracellular ice formation. In MII oocytes these parameters are further confounded by the presence of a fragile cytoskeleton that is resistant to the volumetric excursions commonly associated with equilibrium freezing and a highly temperature sensitive meiotic spindle apparatus ( Saragusty and Arav, 2011 ). These time-dependent sensitivities to chilling to 0°C are evident in MII oocytes from a range of species including mouse ( Pickering and Johnson, 1987 ) rhesus monkey ( Songsasen *et al*., 2002 ) and human ( Pickering *et al*., 1990 ; Zenzes *et al*., 2001 ). Indeed, cooling oocytes to room temperature for even as little as 10 minutes can cause irreversible damage to the meiotic spindle. Furthermore, chilling oocytes to 0°C in the presence of a CPA appears to exacerbate these abnormalities ( Mullen *et al*., 2004 ).

Despite these difficulties, significant advances have been made in our ability to cryopreserve MII oocytes in a range of species. The most convincing data on the efficacy of MII oocyte preservation is undoubtedly derived from human ART. Initial attempts to slow freezing human MII oocytes ( Picton and Chambers, 2009 ) met with only limited success as evidenced by the key statistic of live birth rates per oocyte thawed ( Oktay *et al*., 2006 ). Since then the efficacy of MII oocyte cryopreservation has increased dramatically with live birth rates following human MII oocyte vitrification by the best IVF clinics now being equivalent to those achieved during embryo freezing ( Glujovsky *et al*., 2014 ; Rienzi *et al*., 2017 ). The improved efficiency of human MII preservation can be attributed to the development of optimized vitrification protocols that minimize the cells exposure to the cytotoxic impact of high concentrations of usually two or more CPAs including penetrating CPAs such as ethylene glycol and propanediol and non-penetrating CPAs such as sucrose or trehalose. Oocyte cooling rates during vitrification have also been maximized by the commercial development of specialized vitrification devices for use in ART that support the preservation of individual oocytes or small groups of cells within minimal volumes of cryopreservation reagents. As a result of these advances MII oocyte vitrification is no longer considered an experimental technique in human ART ( Rienzi *et al*., 2017 ) and the approach is regularly used to preserve oocytes for young women who are undergoing infertility treatment, teenage girls or young women who need to safeguard their future fertility before exposure to the ablative cancer treatments, or more controversially, young women who wish to delay childbearing and elect to safeguard their future fertility by oocyte banking (Cobo *et al.*, 2016). Regardless of species, it is clear that the success of oocyte cryopreservation is dependent on oocyte quality, a key parameter that is profoundly and negatively influenced by advancing maternal age ( Goldman *et al*., 2017 ).

While significant improvements have been made in human MII oocyte vitrification these methods have had only a limited impact on oocyte banking in animals over and above the laboratory and livestock species that are used as models for the development and testing of the human ART protocols ( Wildt *et al*., 2010 ). Never-the-less encouraging results for MII vitrification have been obtained in some domestic species such as the cat ( Fernandez-Gonzalez and Jewgenow, 2017 ) as well as some exotic species including for example the Tasmanian devil ( Czarny and Roger, 2010 *)* and Mexican gray wolf ( Boutelle *et al*., 2011 ). While commonalities in the biophysical traits associated with cell freezing between taxonomically-related species may be useful to inform protocol development and support improved post-thaw viability across species, in general the similarities between taxa tend to be overshadowed by the magnitude of species specific differences in the ability of their oocytes to tolerate osmotic and toxic effects of CPA exposure and chilling injury ( Woods *et al*., 2004 ). For example, felid oocytes appear far more tolerant to osmotic changes and cold temperatures than oocytes from bovids, cervids or equids that appear to be more sensitive to cold shock ( Comizzoli *et al*., 2010 , 2012 ). Overall the ability of MII oocytes to survive the freezing and thawing, or cooling and warming processes is highly variable and difficult to predict across species (for review see Comizzoli *et al*., 2012 ). Thus despite the advances that have been made with MII oocyte cryopreservation in humans, fundamental cryobiological investigations and further optimization of oocyte cryopreservation protocols combined with comparative studies across species are needed before MII oocytes cryopreservation can be used efficiently to preserve the fertility of wild or endangered animals, especially where the numbers of oocytes available for preservation are limited.

Some of the difficulties associated with the cryopreservation of MII oocytes may potentially be overcome by the preservation of fully grown, but nuclear immature, germinal vesicle (GV) staged oocytes. However, this option has proved inconsistent and like the MII oocyte preservation detailed earlier, success rates appear to be influenced by species. In addition to rodent and ruminants, successful GV oocyte vitrification has recently been reported in equids ( Ortiz-Escribano *et al*., 2018 ) and domestic cats. The evidence suggests that GV oocytes appear to be more cryo-resistant than their MII counterparts (Comizzoli *et al.*, 2008). Regardless of species, GV oocytes cryopreservation requires the freeze-storage of both the gamete and its supporting compliment of cumulus granulosa cells. The success of GV oocyte cryopreservation is critically dependent on the post-thaw/warming maintenance of the functional integrity of the heterologous gap junctional contacts connecting the cumulus cells to the oocyte. This network of cumulus cells supply the oocyte with vital nutrients and signaling molecules that are essential to drive the cytoplasmic and nuclear maturation of the oocyte to the MII stage. Furthermore, cumulus cells have a discretely different cryopreservation optima compared to oocytes. The loss of gap junctional contacts between the cumulus cell compartment and the GV oocyte is a common casualty of the cryopreservation process such that both the capacity of the oocyte to under go *in vitro* maturation and its subsequent fertile potential and developmental competence are severely compromised. Finally, GV oocyte preservation must be supported by the provision of robust culture environments that support oocyte maturation *in vitro* . Insight into the discrete, species-specific differences in the composition of the culture environment required to drive oocyte maturation will be needed to maximize oocyte quality after cryopreservation.

Regardless of their stage of nuclear maturity secondary oocytes can be harvested in humans and other mammalian species by ultrasound-guided laparoscopy or by laparotomy. Controlled ovarian stimulation with exogenous hormones is frequently used to increase the number of appropriately staged antral follicles at the time of oocyte aspiration. Alternatively, secondary oocytes can be harvested by needle aspiration of antral follicles at any stage of the reproductive cycle following ovariectomy, or as a byproduct of surgical procedures or following the natural mortality of an animal.

## Ovarian tissue preservation

Many of the biophysical challenges associated with preserving secondary oocytes can be avoided by the banking of immature primordial oocytes through ovarian tissue cryopreservation. Indeed, the biological characteristics of primordial oocytes are well suited to preservation since primordial oocytes are relatively undifferentiated and, in the case of humans, are 3-4 fold smaller in diameter than their secondary oocyte counterparts. Importantly, primordial oocytes represent the building blocks of female reproduction and, depending on age, the mammalian ovarian cortex is endowed with thousands to millions of primordial oocytes enclosed within primordial follicles ( Picton, 2001 ). The quantity and quality of the primordial follicles within this ovarian reserve ultimately defines the reproductive longevity and fertile potential of an individual. While our understanding of the mechanisms that regulate the activation of primordial follicle growth and hence depletion of the ovarian reserve is limited, especially in wild animals and there is considerable variability between species, ultimately the ovarian reserve represents the major germplasm resource that can be consistently and safely banked by wildlife reproductive biologists, veterinarians and clinicians alike as a means of safeguarding future fertility and maintaining genetic diversity.

Strategies for ovarian tissue cryopreservation are predominantly centered around the preservation of primordial follicles *in situ* either within fragments of ovarian cortex or by banking the whole ovary. It is possible to freeze-store isolated primordial oocytes but in the absence of supporting ovarian stromal cells these presumptive gametes have only limited viability post thaw/warming. The efficacy of ovarian cryopreservation is dependent on: optimization of the cryopreservation protocol for the species of interest; the individual’s age at the time of tissue harvest and storage; the requirement to successfully preserve both the structural integrity and biological complexity of the tissue. Ovarian cortical tissue is comprised of functionally diverse cell types including immature GV oocytes, follicular somatic cells, stromal cells, blood vessels and the ovarian surface epithelium ( Picton *et al*., 2000 ; Picton, 2001 ). All of these cell types have different cryopreservation optima and all play different but important functional roles in supporting the activation and completion of follicle and oocyte growth and as well as the maintenance of the ovarian reserve, whether *in vivo* or *in vitro*. Finally, strategies designed to successfully preserve the integrity and fertile potential of ovarian tissues and, conversely, to limit cryo-damage need to be developed in parallel with the methods to use the tissue to restore fertility following banking. Despite these challenges ovarian tissue cryopreservation have been optimized and used successfully for a range of species including mice, rats, rabbits, cats, dogs, sheep, goats, cows, pigs, horses, camels, wallabies, wombats, dasyurids, elephants, monkeys and humans (for review see Devi and Goel, 2016 ).

The majority of methods used to cryopreserve ovarian tissue are based on the original validated slow freezing protocols developed for the successful cryopreservation of human ovarian cortex as published by Newton *et al*. (1996) and the associated publications and live births recorded in sheep by Gosden *et al*. (1994) and Baird *et al*. (1999) . In general, ovarian cortex is harvested following ovary removal or ovarian biopsy and thin fragments of cortex are equilibrated in CPA before undergoing controlled rate cooling with manual ice seeding before the temperatures are brought down to -196^o^C and the tissue is stored at liquid nitrogen temperatures. There is now a substantial clinical evidence base to substantiate these early successes. Several thousand girls and young women worldwide have safeguarded their future fertility using ovarian tissue preservation methods. The most common indication for ovarian tissue banking is the need to preserve the future fertility of girls and women: who are at risk of premature ovarian failure (POF) following exposure to the ovotoxic impact of chemotherapy agents for the treatment of cancer ( Gellert *et al*., 2018 ; Oktay *et al*., 2018 ) or other haematological pathologies such as sickle cell anemia ( Demeestere *et al*., 2015 ) or beta thalassemia ( Matthews *et al*., 2018 ); or who carry a high risk of POF for genetic conditions such as Turners syndrome ( Oktay *et al*., 2016 ); or who will suffer ovarian tissue damage and loss following abdominal trauma.

The potential of the stored ovarian tissue to support fertility restoration when the individual wishes to start her family is demonstrated by the delivery of more than 130 healthy babies world wide after autotransplantation of the frozen-thawed tissue and the restoration of endocrine and ovarian function ( Pacheco and Oktay, 2017 ) with more ongoing pregnancies being recorded. Although the overall efficiency of fertility restoration following ovarian tissue preservation and transplantation is unknown, recent evidence from defined cohorts of patients suggests a ~30% live birth rate after autotransplantation of slow frozen-thawed ovarian cortex, ( Jadoul *et al*., 2017 ; Keden *et al.*, 2018). When fertility preservation strategies require the removal of the whole ovary and it is age appropriate to do so, ovarian cortex harvest and cryopreservation can be most effectively combined with methods for *in vitro* maturation and vitrification of MII oocytes to maximize the likelihood of a future successful pregnancy outcome for the patient. Although significant progress has been made in the development and use of ovarian tissue cryopreservation as a means to safeguard the future fertility of girls and young women at risk of POF, further optimization of the cryopreservation and transplantation protocols are likely to be beneficial as the longevity of ovarian autograft function following transplant remains unclear. The latter is likely to be determined by patient age at tissue harvest and by the degree of follicle loss that results from ischaemia and reperfusion injury following ovarian tissue transplantation. Importantly, further research is also needed to define and mitigate against any potential risk of reseeding cancer cells through the transplanted ovarian tissues ( Dolmans and Masciangelo, 2018 ).

While the majority of successful live births have occurred following the slow freezing and autotransplantation of human ovarian tissues, recent evidence from a number of species, including women, suggests that it may also possible to preserve ovarian tissue by vitrification. Histological evaluations and assessment of the viability of oocytes grown *in vitro* from vitrified ovarian tissues have been published for a range of species including, for example, mouse ( Tokieda *et al*., 2002 ; Migishima *et al*., 2003 ), sheep ( Bordes *et al*., 2005 ) cow ( Kagawa *et al*., 2009 ); goat ( Santos *et al*., 2007 , Carvalho *et al*., 2013 ); camel ( Madboly *et al*., 2017 ) dog ( Ishijima *et al*., 2006 ), non-human primate ( Santana *et al*., 2012 ) and human ( Suzuki *et al.,* 2015 ). Collectively these results suggest that it is possible to maintain tissue integrity and oocyte health following super cooling and warming. However, other studies are more skeptical about the suitability of vitrification for ovarian cryopreservation and have recorded increased necrosis in vitrified ovarian tissues ( Rahimi *et al*., 2004 ; Gandolfi *et al*., 2006 ). Overall, at the time of writing it appears that the outcome of ovarian tissue vitrification is far more variable than slow freezing methods and considerable further research effort is needed to optimize and validate vitrification protocols before this approach can be considered a reliable method for the preservation for complex ovarian tissues. Furthermore, the risk of freeze fracture of vitrified samples during long-term tissue storage and compatibility/ potential risk of de-vitrification of samples during tissue bank audits and/or transportation remains to be proven.

An alternative approach than can be used to maximize the preserved ovarian reserve whilst minimizing the potential for ischemic follicle loss following autografting is to cryopreserve and re-transplant the whole ovary. This fertility preservation strategy involves the cryopreservation of not only the intact, whole ovary but also its supporting blood vessels ( Martinez-Madrid *et al*., 2004 ; Baudot *et al*., 2007 ). Evidence to support the concept of the slow freezing and transplantation of whole ovaries and their supporting vasculature has been provided in sheep with the total restoration of natural fertility in all of the treated animals with 64% live birth rate following whole organ preservation and transplantation ( Onions *et al*., 2009 , 2013 ; Campbell *et al*., 2014 ).

## Lessons from human ART and tissue cryopreservation

The development of strategies for human fertility preservation are informing the development of an equivalent agenda in animals. Valuable lessons can be learned from clinical ARTs and human fertility preservation programmes that can be applied to domestic livestock and also be used for the conservation of wild or endangered animals by the cryopreservation of sperm, oocytes and embryos and the preservation of intact gonadal tissues ( Paulson and Comizzoli, 2018 ). Examples of exactly how the potential of bio banks and ARTs such as artificial insemination with cryopreserved sperm have been used successfully to save endangered animals are provided by giant pandas ( Huang *et al*., 2012 ), domestic and wild carnivores ( Comizzoli *et al*., 2009 ; Comizzoli and Wildt, 2012 ) and the black footed ferret ( Howard and Wildt, 2009 ). In contrast to the successful use of sperm freezing, the recent progress made with human oocyte cryopreservation by vitrification has been far more difficult to translate to conservation programmes in animals due to a general lack of basic understanding of the complexity of mature oocyte biology and their cryo-sensitivity in different, target species ( Comizzoli, 2017 ). Further collaborative research effort and cross species comparisons are clearly needed.

The practice of ovarian tissue preservation is highly relevant to the maintenance of the genetic diversity in domestic animals including bovine, ovine, caprine, swine and equine species as well as in wild animals. Indeed, the development of ovarian tissue cryopreservation and fertility restoration strategies in humans have been underpinned by animal models of ovarian freezing. For example, the original proof of principle studies used to establish CPA toxicity and penetrability into ovarian tissues and to determine if the histology and function of these tissues could be retained following slow freezing were conducted in mice ( Harp *et al*., 1994 ) and marmosets ( Candy *et al*., 1995 ) as well as humans ( Newton *et al*., 1998 ). The efficacy of ovarian tissue preservation methods have been evaluated by histological analysis of frozen-thawed tissue and by assessment of follicle and oocyte morphology post thaw/warming in animal tissues, e.g. in cattle ( Gandolfi *et al*., 2006 ) and sheep ( Chambers *et al*., 2010 ) or after short term culture and staining for cell viability and/or apoptotic markers ( Chambers *et al*., 2010 ). Furthermore, while sheep models of ovarian tissue cryopreservation and autografting provided the first definitive proof that cryopreserved ovarian tissue could be used to restore natural fertility with the production of live young in large animals ( Gosden *et al*., 1994 ), live births has previously been reported in laboratory species including mouse (Parrot, 1960), rat ( Wang *et al*., 2002 ) and rabbit ( Almodin *et al*., 2004 ). Similarly, as detailed above the concept of the slow freezing and transplantation of whole ovaries and the supporting ovarian vasculature with the complete restoration of natural fertility has been demonstrated in sheep ( Onions *et al*., 2009 , 2013 ; Campbell *et al*., 2014 ). Other comparisons of the efficacy of whole ovary preservation by slow freezing *vs *. vitrification methods are ongoing across a range of species including mice, goat and cow in the anticipation that these experimental findings can in the future be translated for human use. Finally, xenografting was originally established as model to test the viability ( Oktay *et al*., 1998 ), quality, integrity ( Kim *et al*., 2005 ) and safety of the ovarian tissue transplantation following the preservation of tissue for cancer patients ( Kim *et al*., 2001 ).

While ovarian tissue preservation can clearly be used to preserve and exploit the germplasm of domestic animals of high genetic merit, this technology is also particularly relevant to the preservation and long-term storage of the germplasm of rare or endangered species and/or indigenous native animal breeds where the creation of genetic resource banks is urgently required to maintain future genetic diversity. Over recent years there has been an increase in the number of *ex situ* conservation programmes involving both *in vivo* and *in vitro* preservation and the establishment of germplasm banks ( Silva *et al*., 2012 ). The latter provides a means of connecting *in situ* and *ex situ* conservation programmes ( Andrabi and Maxwell, 2007 ). However it will be difficult to optimize ovarian cryopreservation methods for rare or wild animals which may have highly specialized reproductive adaptations and/or for which only a limited amount of tissue can be safely harvested and cryopreserved. For this group ovarian cryopreservation protocols that have been developed for closely related domestic animals have been adapted and applied to preserve the germplasm of their endangered counterparts ( Leibo and Songsasen, 2002 ). The avoidance of the impact of seasonality on mature oocyte quality and the lack of need for fresh or cryopreserved sperm for IVF at the time of oocyte harvest is a further significant practical advantage of ovarian tissue banking for animals species compared to oocyte or even embryo banking. For occasions where serious damage occurs that results in the unexpected slaughter of a valuable or particularly rare animal (e.g. after limb fracture) multiple ovarian cortical tissue fragments and/or whole ovary(s) can be harvested for storage. Thus cryopreservation strategies developed for, for example, cattle, sheep, goats, horses or pigs, can be used as models for tissue preservation in non-domestic ungulates, while domestic cats can provide valuable models for tissue preservation in wild felids ( Luvoni, 2006 ), dogs for rare canids, and capuchin monkeys for new world primates etc ( Comizzoli and Wildt, 2012 ). Ovarian tissue banking may therefore prove to be a practical and effective tool for the conservation of rare species.

## Fertility restoration following ovarian tissue cryopreservation

A significant and outstanding challenge that needs to be addressed in parallel with the development and optimization of protocols for the banking and long-term storage of the germplasm for humans and domestic animals or wild species, is how best to use the stored tissue to restore fertility and/or generate offspring. Regardless of the origin of the stored ovarian tissue, our ability to realize its potential and produce viable offspring is rigidly linked to our depth of understanding of the reproductive characteristics of the species involved. Fertility restoration and live birth has been achieved in sheep and women where, in both cases, it is possible to autotransplant cryopreserved tissues. The issue of how to realize the potential of preserved ovarian tissues in wild animals or endangered species is far more challenging as autografting may not be feasible. Evidence from a number of species indicate that xenografting of cryopreserved ovarian tissue into mice with severe combined immunodeficiency may provide a temporary solution to this problem. Indeed, xenografting has been shown to support the activation of primordial follicle growth and antral follicle production in cryopreserved ovarian tissues from a range of rodent and domestic species including mouse ( Snow *et al*., 2002 ), cat ( Bosch *et al*., 2004 ), and cow as well as a variety of wild animals including tammar wallaby ( Mattiske *et al*., 2002 ), common wombat ( Cleary *et al*., 2003 ), African elephant, ( Gunasena *et al*., 1998 ) and lion ( Wiedemann *et al*., 2012 ). The combined approach of ovarian tissue banking followed by xenografting may in the future be used in conjunction with follicle culture technologies to produce full sized, MII oocytes for *in vitro* fertilisation and embryo transfer into a suitable recipient animal.

Multiphase culture strategies are being developed to support the complete *in vitro* growth and maturation (IVGM) of oocytes from the primordial follicle stage to maturity in the laboratory ( Picton *et al*., 2008 ). This goal is very ambitious but never-the-less significant advances have been made in our ability to support the IVGM of oocytes in mice ( Eppig and O’Brien 1996 ), sheep ( Newton *et al*., 1999 ; Picton *et al*., 2003 ) cow, (McLaughlin and Telfer, 2010), non-human primates, ( Xu *et al*., 2011 ) and humans (McLaughlin *et al*., 2018). To date the production of healthy, live offspring from primordial follicles has only been achieved in mice ( Eppig and O’Brien, 1996 ; Hikabe *et al*., 2016 ; Hayashi *et al*., 2017 ). It is important to note that on the basis of the published literature it is highly likely that the culture timelines, nutrient requirements needed to support follicle and oocyte growth *in vitro*, the subsequent fertile capacity of the *in vitro* derived oocytes and, ultimately, the efficiency of IVGM strategies for cryobanked tissues will be species specific. Furthermore, it is likely that no single IVGM strategy will fit all species and IVGM strategies for rare or wild species will likely have to be used in conjunction with xenografting in order to realize the fertile potential of the stored germplasm.

## Conclusion

In conclusion although many questions remain to be answered, considerable recent progress in cryobiology, reproductive science, and IVGM technology have led to therapeutic advances in clinical ART that have significantly improved our ability to cryopreserve female fertility by banking primordial oocytes *in situ* within ovarian tissues or by the vitrification of MII oocytes. The stage is now set to translate these clinical advances for animal conservation and to use them to develop comprehensive strategies that will not only safeguard the future genetic diversity of commercially important domestic species but will also facilitate germplasm preservation for animals at risk of extinction.
